# Resveratrol reverses doxorubicin-resistance and alleviates doxorubicin-induced myocardial injury in hepatocellular carcinoma

**DOI:** 10.3389/fcell.2026.1925030

**Published:** 2026-09-09

**Authors:** Shiliang Diao, Shiqi Diao, Mengmeng Wu, Ningning Su, Yinji Quan

**Affiliations:** 1 Special Consultation Clinic of General Internal Medicine, Jilin Provincial People’s Hospital, Changchun, Jilin Province, China; 2 Department of Anesthesiology, Jilin Provincial Cancer Hospital, Changchun, Jilin Province, China; 3 Department of General Practice, Jilin Provincial People’s Hospital, Changchun, Jilin Province, China

**Keywords:** doxorubicin, hepatocellular carcinoma, myocardial injury, oxidative stress, resistance, resveratrol

## Abstract

**Background:**

Doxorubicin (DOX) chemotherapy for hepatocellular carcinoma is hampered by tumor multidrug resistance and cumulative cardiotoxicity. Resveratrol (RES), a natural polyphenol with anti-tumor and antioxidant effects, may reverse DOX resistance and mitigate myocardial injury.

**Objective:**

To investigate whether RES reverses DOX resistance and boosts anti-tumor activity in HepG2-R/SMMC-7721-R cells via apoptosis and EMT regulation, while protecting H9c2 cardiomyocytes; an H22 tumor-bearing mouse model was used to confirm the dual *in vivo* efficacy of combined RES + DOX therapy.

**Methods:**

*In vitro*, CCK-8, morphology observation, wound-healing, Transwell and TUNEL assays detected cell proliferation, migration and apoptosis. Western blot measured apoptosis- and EMT-related proteins. Intracellular ROS, SOD and MDA were tested to clarify antioxidant cardioprotective mechanisms. *In vivo*, tumor volume, body weight, serum myocardial injury biomarkers (CK-MB, cTnI, BNP), oxidative-stress indicators, as well as H&E and Masson´s trichrome staining of myocardial tissue were detected to assess treatment efficacy and safety.

**Results:**

DOX monotherapy barely inhibited resistant cell growth, while RES alone exerted weak anti-tumor activity; RES + DOX exerted obvious combined cytotoxicity to suppress proliferation, metastasis and induce tumor apoptosis. RES reversed EMT and enhanced the activation of Bax/Bcl-2/caspase-3 signaling. In H9c2 cells, RES eliminated DOX-triggered ROS overload, restored SOD, lowered MDA and suppressed cardiomyocyte apoptosis. *In vivo*, the combination achieved optimal tumor suppression, reversed DOX-induced weight loss, normalized serum cardiac-specific damage biomarkers and myocardial oxidative stress. Histological examinations demonstrated that RES alleviated DOX-provoked myocardial structural damage and interstitial fibrosis, and RES itself showed no intrinsic cardiac toxicity.

**Conclusion:**

RES reverses DOX resistance and enhances anti-tumor effects; this finding is associated with the regulation of apoptosis and EMT signaling, and alleviates DOX-mediated cardiomyocyte oxidative injury through antioxidant pathways. This dual-action adjuvant strategy widens DOX´s therapeutic window for DOX-resistant hepatocellular carcinoma.

## 1 Introduction

Liver cancer, a malignant tumor with the highest incidence and mortality rates worldwide, has become a major public health issue threatening human life and health. According to GLOBOCAN 2020 statistics, there are 905,000 new liver cancer cases and 830,000 deaths globally each year, accounting for 9.1% of all cancer-related deaths. Among them, hepatocellular carcinoma accounts for more than 85%, and approximately 70%–80% of patients are diagnosed at advanced stages, missing the critical opportunity for radical surgery (Sung et al., 2021). In recent years, targeted therapies (e.g., sorafenib, lenvatinib) and immune checkpoint inhibitors have achieved breakthrough progress in liver cancer treatment, significantly improving therapeutic outcomes in some patients (Man et al., 2021; De Martin et al., 2025). However, drug resistance remains prevalent throughout clinical treatment, serving as a core bottleneck restricting patients’ survival benefits (Bao et al., 2021). Currently, this therapeutic impasse caused by chemotherapy resistance not only results in an extremely poor prognosis for liver cancer patients, with a 5-year survival rate of less than 15%, but also imposes heavy economic and medical burdens on patients’ families and the broader healthcare system (Xue et al., 2024). Therefore, developing safe and efficient new strategies for reversing drug resistance has become an urgent key task in clinical liver cancer treatment.

Doxorubicin (DOX) is a classic anthracycline chemotherapeutic agent with broad-spectrum anti-tumor activity. It is occasionally applied as a palliative chemotherapy option for advanced or metastatic hepatocellular carcinoma in clinical practice. However, its clinical application in liver cancer is not routine and is greatly limited by inherent drug resistance and cumulative cardiotoxicity (Cao et al., 2023; Kaplan, 2023). Nevertheless, the widespread occurrence of DOX resistance severely impairs its clinical efficacy, becoming the primary obstacle limiting its clinical application. Once drug resistance occurs, it directly leads to a significant increase in tumor recurrence and distant metastasis risks, substantial shortening of patients’ median survival, severe limitation of subsequent treatment options, and a sharp decline in therapeutic efficacy, eventually trapping patients in a dilemma of no available drugs (Elfiky et al., 2025). Meanwhile, the dose-dependent cumulative cardiotoxicity of DOX is another major clinical limitation with irreversible potential harm; even low-dose administration may induce subclinical myocardial injury, seriously reducing patients’ treatment tolerance and impairing long-term quality of life (Christidi and Brunham, 2021). Such cardiotoxicity may not only force clinicians to reduce the DOX dosage or even terminate chemotherapy, but also trigger persistent cardiac dysfunction, further exacerbating poor prognosis and seriously affecting patients’ survival benefits (Sheibani et al., 2022). Thus, how to effectively reverse liver cancer cell resistance to DOX, enhance its antitumor efficacy, and specifically antagonize its cardiotoxicity to achieve the triple therapeutic goals of resistance reversal, efficacy enhancement, and toxicity reduction has become a critical scientific problem to be addressed urgently in liver cancer chemotherapy, which holds great clinical value and practical significance for improving therapeutic effects and quality of life in DOX-resistant liver cancer patients. Clinically, dexrazoxane is the only approved cardioprotective agent for anthracycline-induced cardiotoxicity. However, its application is limited due to potential interference with chemotherapy efficacy and partial protective insufficiency. Therefore, developing novel agents that simultaneously reverse chemotherapy resistance and reduce cardiotoxicity without compromising antitumor activity is urgently needed.

Natural active ingredients have shown enormous development potential and application prospects in chemotherapy sensitization, toxicity reduction and drug resistance reversal for tumors due to their wide sources, abundant pharmacological activities and low toxic and side effects, emerging as a research hotspot in current tumor pharmacology. Resveratrol (RES) is a natural polyphenolic active component widely existing in grapes, peanuts, Polygonum cuspidatum and other plants. Modern pharmacological studies have confirmed its multi-target and multi-pathway biological activities, including anti-tumor, anti-inflammatory, anti-oxidative, anti-apoptotic and multi-organ protective effects, with good biosafety in preclinical studies, while its clinical transformation is still restricted by pharmacokinetic defects (Ashrafizadeh et al., 2021; Cui et al., 2022; Taguchi et al., 2016; Ma et al., 2023). In liver cancer-related studies, numerous studies have verified that RES exerts significant anti-tumor effects by inhibiting liver cancer cell proliferation, arresting cell cycle progression, inducing tumor cell apoptosis, and suppressing tumor cell migration and invasion, showing favorable therapeutic potential for liver cancer (Wang P. et al., 2024). More importantly, existing studies suggest that RES has potential chemoresistance reversal activity, which can enhance the sensitivity of drug-resistant tumor cells to chemotherapeutic agents, providing a new research direction and idea for solving chemoresistance problems (Xu et al., 2017). In addition, RES exhibits significant cardioprotective effects in various animal and cell models of myocardial injury, effectively alleviating myocardial cell damage and improving cardiac function, serving as a potential candidate drug for antagonizing chemotherapy-induced cardiotoxicity (Wang Y. et al., 2024).

Although existing studies have preliminarily confirmed the multiple potentials of RES in anti-tumor, chemoresistance reversal and cardioprotection, systematic research on its application in DOX-resistant liver cancer treatment remains insufficient, with unclear efficacy and molecular mechanisms. Specifically, it remains unclarified whether RES can reverse the DOX-resistant phenotype of hepatocellular carcinoma (HCC) cells and exert differential regulatory effects on tumor cells and cardiomyocytes to achieve chemo-sensitization and cardioprotection simultaneously. Herein, we systematically validated the dual functions of RES in reversing DOX resistance and alleviating DOX-induced myocardial injury via *in vitro* cell models and *in vivo* H22 tumor-bearing mouse models. A multi-dimensional evaluation system was adopted to elaborate the core mechanism of RES, aiming to provide reliable experimental evidence and theoretical reference for optimizing chemotherapy regimens for DOX-resistant hepatocellular carcinoma.

## 2 Materials and methods

### 2.1 Cell culture and treatment

DOX-resistant HCC cell lines (HepG2-R and SMMC-7721-R) were successfully established via continuous and intermittent induction with gradually increasing concentrations of DOX over more than 6 months. Briefly, parental HepG2 and SMMC-7721 cells were exposed to stepwise elevated DOX concentrations (0.1–2 μM) with intermittent drug withdrawal culture to screen and obtain stably resistant cell phenotypes. To strictly verify the successful construction of drug-resistant cell models, we detected the DOX half-maximal inhibitory concentration (IC_50_) of parental cells and induced resistant cells by CCK-8 assay, and calculated the resistance index (RI) to evaluate the stability and resistance level of cell lines. The calculation formula of RI was as follows: RI = IC_50_ (resistant cell line)/IC_50_ (parental cell line).

The quantitative results showed that the IC_50_ values of DOX for parental HepG2 and SMMC-7721 cells were 1.86 μM and 2.03 μM, respectively. In contrast, the IC_50_ values of DOX for induced HepG2-R and SMMC-7721-R resistant cells were significantly increased to 12.98 μM and 14.21 μM, respectively. Correspondingly, the RI values of HepG2-R and SMMC-7721-R cells reached 6.98 and 7.00, respectively. A RI value greater than 6.0 indicates that the cell lines have obtained stable and high-level DOX resistance. The above data fully confirmed that the two resistant cell lines constructed in this study had stable and reliable DOX-resistant phenotypes, which met the experimental requirements of subsequent drug resistance reversal and mechanism verification experiments.

Human hepatocellular carcinoma cell line HepG2, its DOX-resistant subline HepG2-R, human hepatocellular carcinoma cell line SMMC-7721, its DOX-resistant subline SMMC-7721-R, and rat cardiomyocyte cell line H9c2 were routinely cultured *in vitro* in high-glucose DMEM supplemented with 10% fetal bovine serum (FBS) and 1% penicillin-streptomycin. Cells were maintained in a humidified incubator at 37 °C with 5% CO_2_. When cell confluence reached 80%–90% for routine subculture, adherent cells were digested with 0.25% trypsin-EDTA for subculture. All cell lines were seeded at appropriate densities in corresponding 6-well plates, 96-well plates and confocal dishes 24 h prior to experiments to ensure cells were in the logarithmic growth phase with favorable physiological status during formal experiments. RES stock solution was prepared with dimethyl sulfoxide (DMSO), and DOX stock solution was dissolved in phosphate-buffered saline (PBS, pH = 7.4); both were serially diluted to preset working concentrations with complete medium before cell treatment. The final concentration of DMSO in the solvent control group was strictly controlled below 0.1% to avoid non-specific cytotoxicity induced by organic solvents and ensure experimental accuracy.

### 2.2 Cell proliferation assay

Cell proliferation was quantitatively determined using the cell counting kit-8 (CCK-8). DOX-resistant hepatocellular carcinoma cells (HepG2-R, SMMC-7721-R) were seeded in 96-well plates at a density of 5,000 cells per well. After 24 h of adherent culture at 37 °C with 5% CO_2_, cells were treated with gradient concentrations of DOX, RES and their combination, with three biological replicates per group to reduce experimental error. At 24 h and 48 h post-treatment, the medium was discarded, and 100 μL of fresh serum-free DMEM containing 10% CCK-8 solution was added to each well, followed by incubation at 37 °C with 5% CO_2_ for 30 min. The absorbance (OD value) at 450 nm was measured using a microplate reader. Relative cell viability was calculated against the solvent control group using the formula: Cell viability=(OD value of treatment group/OD value of control group) × 100%. H9c2 cardiomyocytes were pre-treated with RES at preset concentrations for 2 h, then co-cultured with DOX for 24 h, with subsequent CCK-8 detection steps consistent with those for hepatocellular carcinoma cells. All proliferation experiments were independently repeated 3 times under sterile conditions.

### 2.3 Cell morphological analysis

For cell morphological analysis, DOX-resistant hepatocellular carcinoma cells (HepG2-R, SMMC-7721-R) were seeded in 6-well plates at a density of 1 × 10^5^ cells per well. Cells were cultured in complete medium until reaching 80% confluence before drug treatment. Cells were seeded and maintained in complete medium alone (serving as the negative control [NC]), or in medium supplemented with DOX (DOX group), RES (RES group), or a combination of both agents (Com group). After 24 h of continuous culture, the medium was discarded, and cells were gently washed twice with pre-cooled PBS to remove residual medium. Cell morphology was observed and photographed under an inverted phase-contrast microscope; cell adherence ability and intracellular granule changes were recorded, and morphological differences between different treatment groups and the blank control group were systematically compared.

### 2.4 Cell migration assay

Wound healing assay and Transwell assay were combined to evaluate the *in vitro* migration ability of DOX-resistant hepatocellular carcinoma cells (HepG2-R, SMMC-7721-R), with mutual verification to ensure result reliability. For wound healing assay: logarithmic-phase DOX-resistant hepatocellular carcinoma cells were seeded in 6-well plates and cultured to form dense confluent monolayers. Uniform linear scratches were made on cell monolayers with a sterile 200 μL pipette tip along a ruler. Detached cells and debris were gently washed away with PBS, and cells were treated with serum-free medium containing RES, DOX or RES + DOX. The plates were placed in a humidified incubator at 37 °C with 5% CO_2_, and cell migration and wound healing were observed at 24 h and 48 h. Images of scratch areas were captured under a microscope; scratch width changes were measured via ImageJ software to calculate wound healing rate for quantitative evaluation of cell migration rate.

For Transwell assay: 200 μL of cell suspension (1 × 10^6^ cells/mL) prepared with serum-free medium was added to the upper chamber, and complete medium containing RES, DOX or RES + DOX was added to the lower chamber as a chemoattractant. After 24 h of culture, non-migrated cells on the upper membrane were wiped off with a sterile cotton swab, and the assay was completed at 48 h. Migrated cells that passed through the membrane were fixed with 4% paraformaldehyde at room temperature for 30 min and stained with 0.1% crystal violet. Stained cells were counted in five randomly selected fields under an optical microscope, and the average value was used to quantify migration ability. All migration experiments were performed under sterile conditions and independently repeated 3 times. For migration assay, no Matrigel was coated on the Transwell membrane. Of note, the invasion assay was not performed in the present study.

### 2.5 Detection of apoptosis

Terminal deoxynucleotidyl transferase dUTP nick end labeling (TUNEL) assay was used for specific analysis to accurately evaluate cell apoptosis under different drug treatments, which directly labels DNA breakage sites of apoptotic cells. Logarithmic-phase DOX-resistant hepatocellular carcinoma cells (HepG2-R, SMMC-7721-R) and H9c2 cardiomyocytes were seeded in confocal dishes at a density of 2 × 10^4^ cells per well, and cultured at 37 °C with 5% CO_2_ for 24 h to ensure sufficient cell adherence and growth. Hepatocellular carcinoma cells were directly treated with RES or DOX at preset concentrations for 24 h; for the myocardial protection group, H9c2 cells were pre-treated with RES for 2 h followed by co-culture with DOX for 24 h to explore the protective effect of RES against DOX-induced myocardial injury. After treatment, cells were washed 3 times with pre-cooled PBS and stained according to the standard protocol of the TUNEL fluorescence staining kit. Stained cells were immediately observed under a laser confocal fluorescence microscope; apoptotic cells exhibited specific green fluorescence. Cells were counted in five random non-overlapping fields to calculate the ratio of apoptotic cells to total cells for apoptosis rate evaluation. All experiments were performed in dark conditions and independently repeated 3 times, providing data support for subsequent investigation of RES mechanism of action.

### 2.6 Western blot analysis

Western blot was performed to detect the expression levels of key functional proteins in cells, so as to further explore the molecular regulatory mechanisms of DOX-resistant hepatocellular carcinoma cells (HepG2-R) and cardiomyocytes under different drug treatments. All Western blot experiments were independently repeated with three biological replicates and three technical replicates to ensure experimental reproducibility and result reliability. Drug-treated cells were washed 3 times with pre-cooled PBS, then fully lysed on ice with radioimmunoprecipitation assay (RIPA) lysis buffer containing protease and phosphatase inhibitors to extract total cellular proteins. The cell lysate was incubated on ice for 15 min, and cell debris was removed by low-temperature high-speed centrifugation (12,000 rpm, 4 °C, 15 min); the supernatant was collected as total protein samples. Total protein concentration was accurately quantified by bicinchoninic acid (BCA) assay. According to the quantification results, 40 μg of protein samples were mixed with 5×loading buffer and denatured in boiling water at 100 °C for 5 min, followed by sodium dodecyl sulfate-polyacrylamide gel electrophoresis with separation gel concentration of 10%–12% based on the molecular weight of target proteins. After electrophoresis, proteins were transferred onto polyvinylidene difluoride (PVDF) membrane by wet transfer at a constant voltage of 100 V for 1 h under ice bath conditions. The PVDF membrane was blocked with 5% skimmed milk at room temperature for 1 h to reduce non-specific binding, then incubated with primary antibodies overnight at 4 °C on a low-speed shaker.

All primary antibodies used in this study were purchased from Abcam (Cambridge, UK) with verified application validity and clear catalog numbers: Bax (ab32503), Bcl-2 (ab182858), cleaved-caspase-3 (ab32042), cleaved-PARP (ab32561), E-cadherin (ab40772), N-cadherin (ab76011), vimentin (ab92547), Snail (ab53519), Slug (ab27568), Tubulin (ab7291). All antibodies have been strictly verified for specificity and effectiveness in human hepatocellular carcinoma cells and rat cardiomyocyte cell lines by the manufacturer, and the antibody application range covers cell Western blot experiments, which meets the experimental requirements of this study. The secondary antibody (HRP-conjugated goat anti-rabbit IgG, ab6721) was diluted at 1:5,000. The membrane was washed 3 times with TBST washing buffer for 5 min each time to remove unbound primary antibodies, then incubated with HRP-conjugated secondary antibodies at room temperature for 1 h on a shaker.

For multi-protein detection of the same PVDF membrane, the standard membrane stripping and re-incubation procedure was strictly performed: after completing the detection of one target protein, the PVDF membrane was immersed in commercial Western blot stripping buffer for 15 min at room temperature to completely strip the bound primary and secondary antibodies. After stripping, the membrane was fully washed with TBST for 3 times (5 min each time), re-blocked with 5% skimmed milk for 30 min, and then incubated with another primary antibody overnight at 4 °C. The above stripping and re-incubation steps were followed for all sequential protein detections to ensure accurate detection of multiple target proteins on the same membrane. Protein signals were visualized using an enhanced chemiluminescence (ECL) chemiluminescence system, and captured with a gel imaging system. Gray values of protein bands were analyzed by Image Lab software, and the gray value ratio of target proteins to internal reference Tubulin was calculated to reflect the relative expression levels of target proteins.

In this study, all complete uncropped original protein band images corresponding to the experimental results have been sorted and retained, including full-length PVDF membrane original scanning pictures of all target proteins and internal reference proteins. All original raw images without any cropping, splicing or modification can be provided to the journal for inspection at any time to ensure the authenticity and repeatability of experimental data. All experiments were independently repeated 3 times, providing evidence for clarifying the related molecular mechanisms.

### 2.7 Detection of oxidative stress markers in cardiomyocytes

Systematic quantitative detection of oxidative stress levels in H9c2 cardiomyocytes was conducted, with three core markers including intracellular reactive oxygen species (ROS) level, superoxide dismutase (SOD) activity and malondialdehyde (MDA) content, to comprehensively reveal the oxidative stress status of cardiomyocytes after drug treatment. Intracellular ROS level was detected using 2′,7′-dichlorodihydrofluorescein diacetate (DCFH-DA) green fluorescent probe. Drug-treated H9c2 cells were incubated with 10 μM DCFH-DA probe at 37 °C in the dark for 30 min, then washed 3 times with pre-cooled PBS to remove free probes. Fluorescence intensity was quantitatively analyzed under a laser confocal fluorescence microscope with excitation wavelength of 488 nm and emission wavelength of 525 nm, to reflect intracellular ROS production. SOD activity was measured by water-soluble tetrazolium salt-8 (WST-8) assay: total intracellular enzyme solution was extracted, mixed with WST-8 working solution and incubated at room temperature for 20 min; absorbance at 450 nm was detected, and SOD activity was expressed as U/mg protein to reflect the scavenging capacity of cells against superoxide anion radicals. MDA content was determined by thiobarbituric acid assay: cell lysate was mixed with thiobarbituric acid (TBA) reagent, heated in a constant temperature water bath at 95 °C for 60 min, then cooled to room temperature in an ice water bath and centrifuged to collect the supernatant. Absorbance at 532 nm was measured, and MDA concentration was calculated via standard curve method, which directly reflects the degree of cell membrane lipid peroxidation damage. All marker detection experiments were independently repeated 3 times.

### 2.8 *In vivo* anti-tumor and myocardial protection experiments

A subcutaneous syngeneic H22 hepatocellular carcinoma mouse model was established to simultaneously evaluate the *in vivo* antitumor potency and myocardial protective capacity of monotherapy and combined RES-DOX treatment regimens. Kunming mice aged 6–8 weeks were raised in a standardized animal room with constant temperature (22 °C ± 2 °C), humidity (50% ± 5%), *ad libitum* access to food and water, and a 12 h light/dark cycle. Mice were subcutaneously inoculated with 5 × 10^6^ logarithmic-phase H22 hepatocellular carcinoma cell suspension (0.1 mL) at the right flank. When the average tumor diameter reached approximately 5 mm, all tumor-bearing mice were randomly assigned to four experimental groups: NC group, RES monotherapy group, DOX monotherapy group, and RES + DOX combined treatment (Com) group.

All therapeutic interventions were delivered via intraperitoneal (i.p.) injection once every 3 days over a continuous 14-day treatment cycle. The dosing regimen was formulated based on our preceding *in vitro* cellular data and previously reported safe *in vivo* doses: the NC group received an equal volume of normal saline; the RES group was injected i. p. With RES (50 mg/kg); the DOX group received i. p. DOX (2 mg/kg); the Com group was co-administered RES (50 mg/kg) and DOX (2 mg/kg) via intraperitoneal injection.

Throughout the full 14-day treatment window, mouse body weight and tumor dimensions were dynamically and synchronously recorded to track tumorigenic progression and systemic physiological changes induced by different treatments. Vernier calipers were used to measure the long and short diameters of subcutaneous tumors, and tumor volume was calculated with the standard formula: Tumor Volume = 0.5 × long diameter × (short diameter)^2^. Continuous body weight monitoring was performed to assess whole-animal physiological status and drug-related systemic toxicity, which served as a direct indicator of treatment tolerability.

Twenty-four hours following the final i. p. Administration, mice were anesthetized with pentobarbital sodium. Blood samples were collected from the abdominal aorta, and serum was separated. Serum myocardial injury biomarkers including creatine kinase-MB isoenzyme (CK-MB), lactate dehydrogenase (LDH), glutamic-oxaloacetic transaminase (GOT), cardiac troponin I (cTnI) and brain natriuretic peptide (BNP) were measured. An automated biochemical analyzer was used for CK-MB, LDH and GOT detection; cTnI and BNP were quantified using corresponding commercial ELISA kits according to manufacturer protocols. For myocardial oxidative stress assessment, heart tissues were rapidly isolated, rinsed with pre-cooled PBS to remove residual blood, and prepared into 10% myocardial tissue homogenates under ice-bath conditions to prevent loss of endogenous enzyme activity. SOD activity and MDA content in tissue homogenates were quantified following the identical experimental protocols described in the *in vitro* section.

For histopathological evaluation, myocardial tissues were fixed in 4% paraformaldehyde, embedded in paraffin and sectioned. Hematoxylin-eosin (H&E) staining was performed for morphological observation, and semi-quantitative myocardial injury scores were calculated. Masson’s trichrome staining was applied to evaluate myocardial interstitial fibrosis; collagen volume fraction (CVF%) was analyzed for quantitative fibrosis assessment.

All animal handling, cell inoculation, drug administration and sample detection procedures were strictly implemented in compliance with the experimental guidelines approved by the Animal Ethics Committee of Jilin Provincial People’s Hospital. This animal study was performed in strict accordance with the ARRIVE 2.0 (Animal Research: Reporting o*f In Vivo* Experiments) guidelines, and all animal experimental operations followed the 3 R principles (Replacement, Reduction, Refinement) of animal experimentation, guaranteeing complete ethical compliance and reliable, reproducible experimental results, and providing solid *in vivo* experimental evidence to support the combinatorial application of RES and DOX for hepatocellular carcinoma treatment.

## 3 Results

### 3.1 Assessment of tumor cell proliferation and morphological changes

**FIGURE 1:**
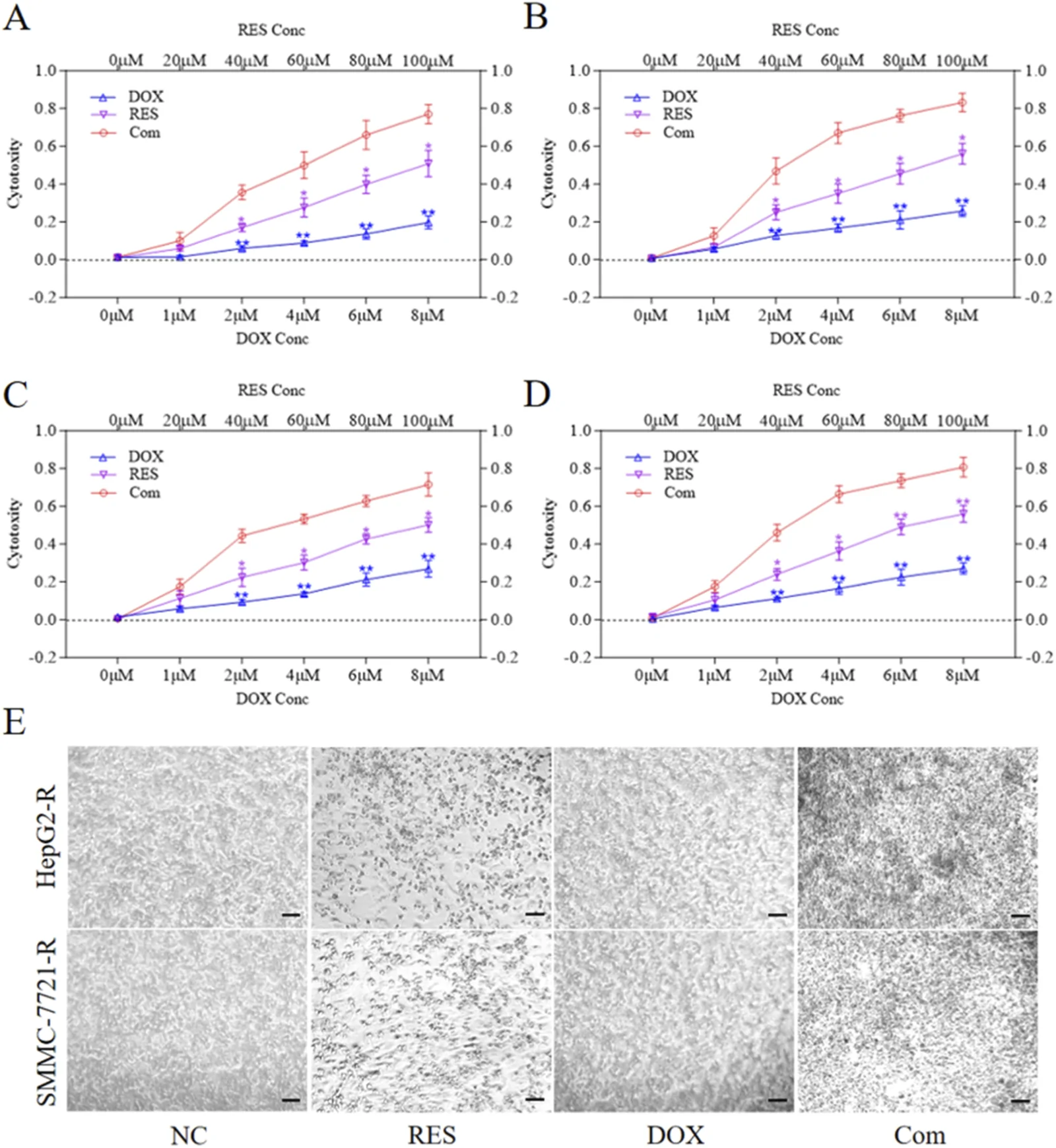
RES and DOX exert an efficacy-enhancing combined effect in eliminating drug-resistant liver cancer cells. **(A–D)** HepG2-R **(A,B)** and SMMC-7721-R **(C,D)** cells were treated with increasing concentrations of RES, DOX, or their combination (Com) for 24 h **(A,C)** or 48 h **(B,D)**. Cell viability was evaluated via CCK-8 assay. All *in vitro* experiments were performed in biological triplicates. Statistical analysis was conducted using one-way ANOVA followed by Tukey´s multiple comparisons test. **(E)** Microscopic observation of morphological changes in HepG2-R and SMMC-7721-R cells after 24 h treatment with 40 μM RES and/or 2 μM DOX. NC: Negative control; RES: Resveratrol; DOX: Doxorubicin; Com: RES + DOX. Scale bar: 100 μm *p < 0.05, **p < 0.01 vs. NC group; #p < 0.05 vs. Com group.

Prior to the formal drug intervention experiment, we strictly verified and supplemented the drug resistance characteristics of DOX-resistant HCC cell lines as required. We comprehensively detected and compared the DOX IC_50_ values of parental HepG2, SMMC-7721 cells and their corresponding resistant sublines, and calculated the RI for quantitative evaluation of cell resistance level. The results showed that the IC_50_ values of DOX in resistant cells were significantly higher than those in parental cells, and the RI values of the two resistant cell lines were both greater than 6.5, confirming the stable and high-level DOX-resistant phenotype of the constructed cell models (Table 1). Consistent with the resistance identification results, subsequent CCK-8 assay results demonstrated that DOX, at specific concentrations, exerted no significant inhibitory effect on the proliferation of HepG2-R (Figures 1A,B) or SMMC-7721-R (Figures 1C,D) cells following incubation for either 24 h (Figures 1A,C) or 48 h (Figures 1B,D). In contrast, RES exhibited a pronounced concentration-dependent cytotoxic effect on DOX-resistant liver cancer cells. Furthermore, the combination of DOX and RES resulted in significantly greater cytotoxicity compared to the single-agent treatments. These findings suggest that RES enhances the cytotoxic efficacy of DOX in DOX-resistant liver cancer cells. Notably, the CCK-8 assay revealed that the efficacy-enhancing cytotoxic effect of 40 μM RES and 2 μM DOX on both HepG2-R and SMMC-7721-R cells was already evident at the 24 h time point. Consequently, in subsequent experiments, the 24 h time point was predominantly selected, with the RES concentration fixed at 40 μM and the DOX concentration maintained at 2 μM.

**TABLE 1:** IC_50_ values and resistance index of DOX in parental and resistant HCC cell lines.

Cell line	DOX IC_50_ (μM)	Resistance index (RI)
HepG2 (parental)	1.86	-
HepG2-R (resistant)	12.98	6.98
SMMC-7721 (parental)	2.03	-
SMMC-7721-R (resistant)	14.21	7.00

As shown in Figure 1E, observations under an inverted bright-field microscope revealed significant differences in morphology, cell density and optical properties of HepG2-R and SMMC-7721-R cells among different treatment groups. Cells in the NC and DOX groups maintained a normal morphology with strong adhesion and abundant cell numbers, and no abnormal deposits were observed in the field of view. In the RES group, partial cells exhibited morphological abnormalities and weakened adhesion capacity, appeared in a suspended state, and were accompanied by granular deposits caused by cell injury and metabolic disorder. The Com group showed a marked reduction in cell adhesion, with a large number of cells suspended and detached, and a prominent increase in granular deposits. These results directly reflected the combined efficacy-enhancing cytotoxic effect of combined RES and DOX treatment on DOX-resistant hepatocellular carcinoma cells, which was consistent with the aforementioned CCK-8 assay results.

### 3.2 Evaluation of cell migration ability

**FIGURE 2:**
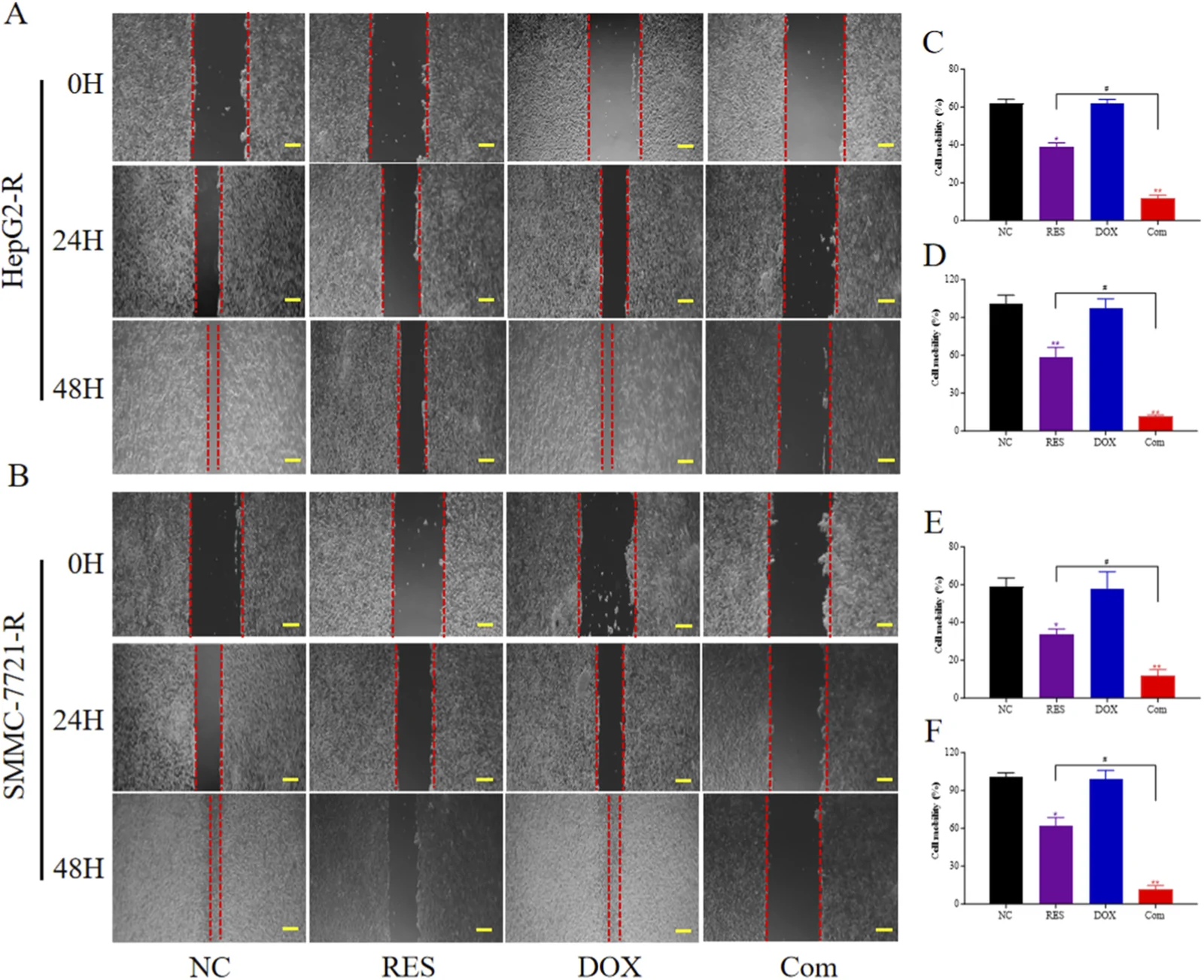
RES and DOX inhibit the migratory ability of drug-resistant liver cancer cells. **(A,B)** Representative scratch wound healing assay images of HepG2-R **(A)** and SMMC-7721-R **(B)** cells at 0 h, 24 h, and 48 h after treatment with 40 μM RES, 2 μM DOX, or their combination. **(C–F)** Quantitative analysis of wound healing rate. All experiments were repeated three biologically independent times. One-way ANOVA with Tukey´s *post hoc* test was used for statistical analysis. NC: Negative control; RES: Resveratrol; DOX: Doxorubicin; Com: RES + DOX. Scale bar: 100 μm *p < 0.05, **p < 0.01 vs. NC group; #p < 0.05 vs. Com group.

When liver cancer patients develop metastasis, the therapeutic challenge increases substantially. Tumor metastasis is highly dependent on cell migration, and suppression of tumor cell migratory capacity plays a critical role in mitigating the adverse effects of metastatic progression. In this study, we assessed cell migration activity using both scratch wound healing and Transwell migration assays. Notably, the combined drug concentration (40 μM RES +2 μM DOX) adopted in this study exerts significant cytotoxicity and proliferative inhibition on resistant HCC cells, accompanied by partial cell detachment and reduced cell adherence. This serves as an important confounding factor for migration phenotypic detection. The results demonstrated that RES effectively slowed the re-attachment rate of HepG2-R and SMMC-7721-R cells into the wounded area. In contrast, DOX exhibited minimal impact on cell motility. The decreased wound healing rate and reduced number of migrated cells in the RES + DOX group were not solely attributed to specific inhibition of cell migration, but represented a comprehensive outcome of drug-induced cell detachment, proliferative arrest, and EMT-mediated specific migration suppression. RES significantly enhanced the anti-migratory effect in DOX-resistant hepatocellular carcinoma cells (Figure 2). The Transwell assay results further corroborated these findings.

**FIGURE 3:**
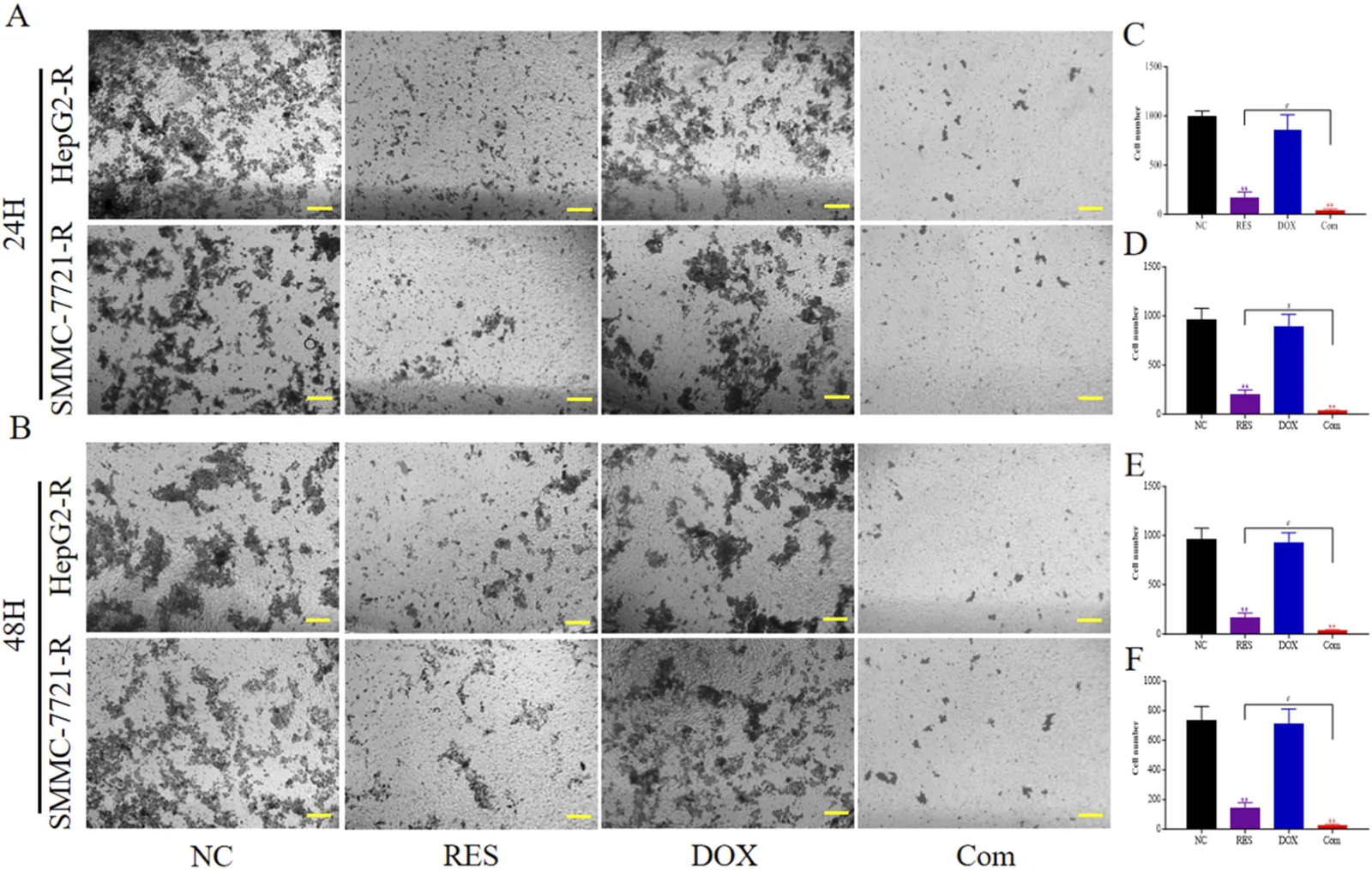
RES and DOX inhibit the migratory ability of drug-resistant liver cancer cells. **(A,B)** Representative Transwell migration assay images of HepG2-R **(A)** and SMMC-7721-R **(B)** cells after 24 h and 48 h treatment. **(C–F)** Quantitative statistics of migrated cell numbers. Data were obtained from three biological replicates. Statistical differences were analyzed by one-way ANOVA followed by Tukey´s test. NC: Negative control; RES: Resveratrol; DOX: Doxorubicin; Com: RES + DOX. Scale bar: 50 μm*p < 0.05, **p < 0.01 vs. NC group; #p < 0.05 vs. Com group.

**FIGURE 4:**
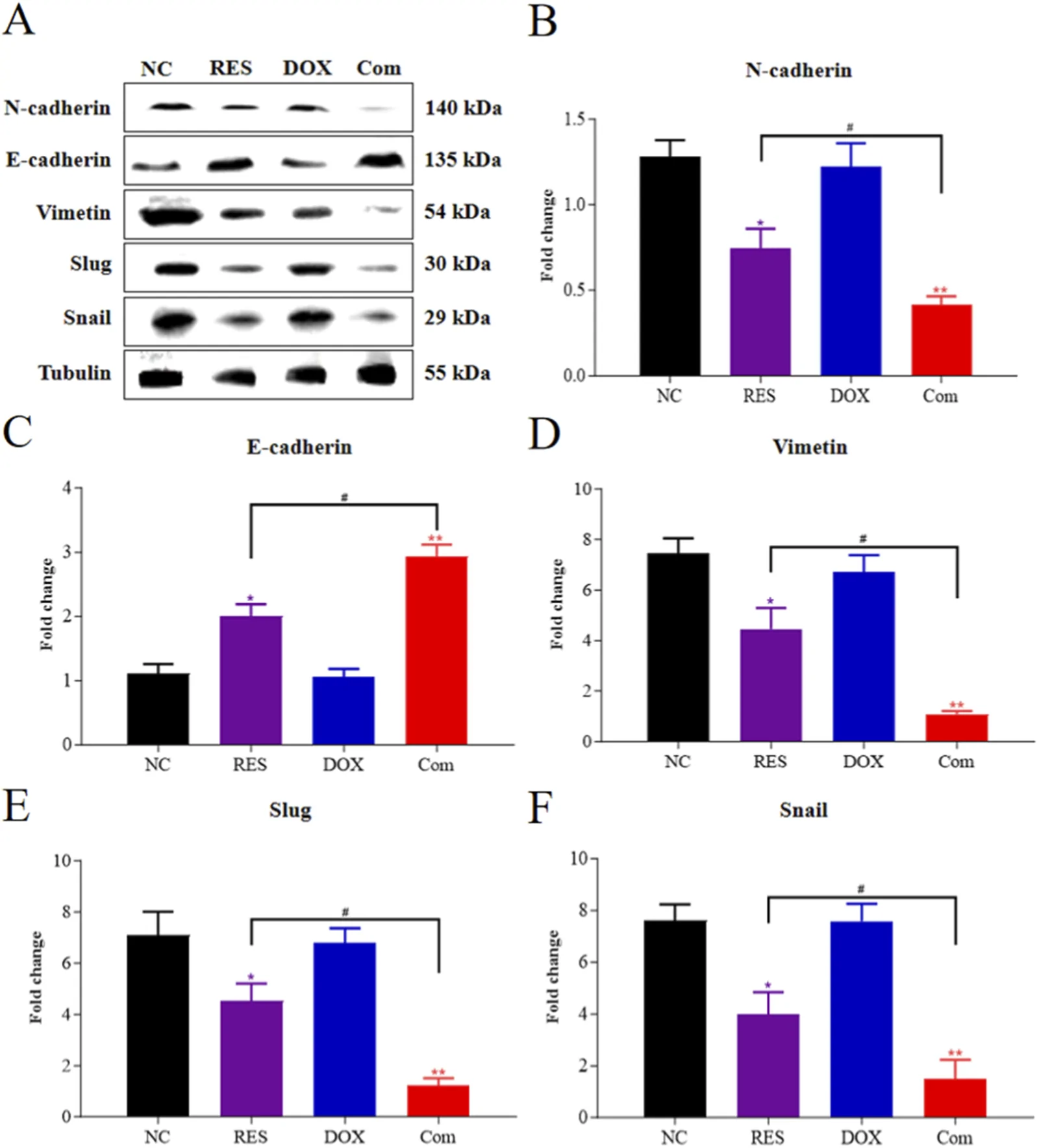
RES and DOX regulate EMT-related protein expression in drug-resistant liver cancer cells. **(A)** Representative Western blot images of N-cadherin, E-cadherin, Vimentin, Slug, and Snail. Tubulin was used as a loading control. **(B–F)** Quantitative analysis of relative protein expression. All Western blot experiments were performed in triplicate biological repeats. One-way ANOVA and Tukey´s multiple comparison test were applied for statistical analysis. NC: Negative control; RES: Resveratrol; DOX: Doxorubicin; Com: RES + DOX. *p < 0.05, **p < 0.01 vs. NC group; #p < 0.05 vs. Com group.

Compared with the NC group, the number of cells migrating through the polycarbonate membrane was markedly reduced in both the RES and Com groups, with the Com group showing the lowest migratory capacity. In contrast, no significant difference in cell migration was observed between the DOX group and the NC group (Figure 3). Furthermore, Western blot analysis was performed to detect the expression levels of key migration-related proteins in HepG2-R cells. The results showed that compared with the NC group, the expression of E-cadherin was significantly upregulated in the RES monotherapy group, while the expression of N-cadherin, vimentin, snail and slug was markedly downregulated. Notably, the RES-DOX combined treatment group (Com group) exhibited more pronounced changes in the above proteins: the expression level of E-cadherin was higher than that in the RES monotherapy group, whereas the levels of N-cadherin, vimentin, snail and slug were all lower than those in the RES monotherapy group, presenting the strongest regulatory effect among all experimental groups (Figure 4).

Overall, these results confirmed that RES could effectively reverse the epithelial-mesenchymal transition (EMT) process in HepG2-R cells by regulating the expression of EMT pathway-related proteins, indicating a specific molecular basis for migration inhibition. Despite the phenotypic confounding caused by drug cytotoxicity and cell detachment, the consistent EMT protein remodeling at the molecular level verified that RES possesses intrinsic activity to suppress HCC migration and metastasis, thereby enhancing the antimigratory effect of DOX on drug-resistant hepatocellular carcinoma cells. Notably, alterations in EMT-related protein expression represent correlative molecular readouts in our system; these observations cannot exclusively prove that EMT reversal serves as the direct causal mechanism underlying RES-mediated chemosensitization.

### 3.3 Analysis of tumor cell apoptosis

**FIGURE 5:**
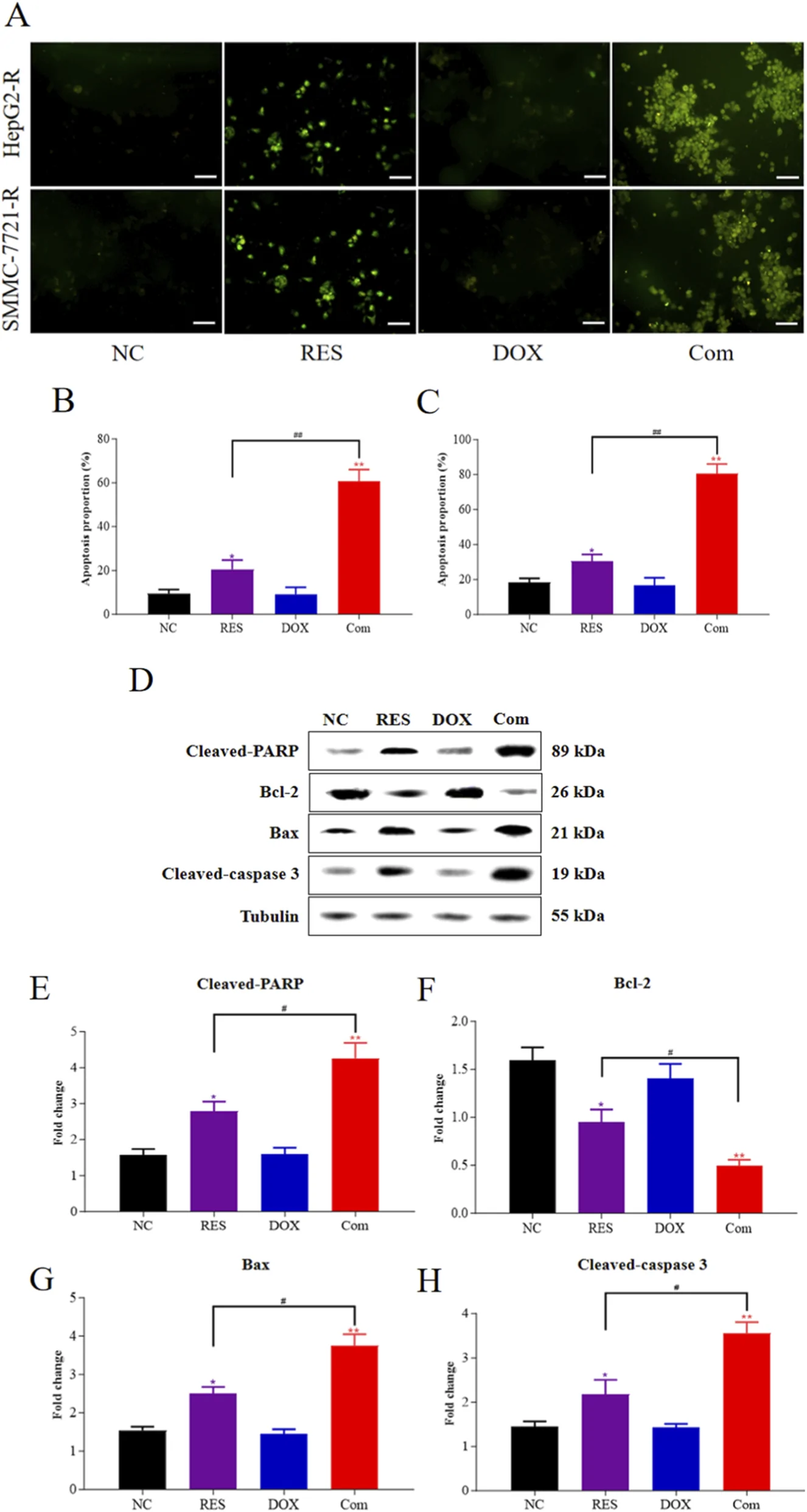
RES and DOX jointly enhance apoptosis in drug-resistant liver cancer cells. **(A)** Representative TUNEL fluorescence staining images of HepG2-R and SMMC-7721-R cells. **(B,C)** Statistical analysis of cell apoptotic rate. **(D)** Representative Western blot images of apoptosis-related proteins. **(E–H)** Quantitative analysis of relative protein levels. All experiments were conducted with three biological replicates. Statistical analysis was performed using one-way ANOVA with Tukey´s *post hoc* test. NC: Negative control; RES: Resveratrol; DOX: Doxorubicin; Com: RES + DOX. Scale bar: 50 μm *p < 0.05, **p < 0.01 vs. NC group; #p < 0.05, ##p < 0.01 vs. Com group.

The apoptotic status of cells was assessed using the TUNEL assay. Under fluorescence microscopy, apoptotic cells exhibited green fluorescence due to the incorporation of labeled dUTP, whereas non-apoptotic cells showed no fluorescent signal. Compared with the NC group, the number of green fluorescent cells was significantly increased in both the RES group and the Com group. Notably, the Com group displayed the highest number of fluorescent cells among all four groups. In contrast, there was no significant difference in the number of green fluorescent cells between the NC group and the DOX group (Figures 5A–C). These findings indicate that RES can induce apoptosis in DOX-resistant liver cancer cells and enhance the pro-apoptotic efficacy of DOX. Western blot analysis was performed to evaluate the expression levels of apoptosis-related proteins in liver cancer cells. Compared with the NC group, the RES group showed a significant upregulation in the expression of cleaved-caspase-3, Bax, and cleaved-PARP. Conversely, the expression of Bcl-2 was markedly downregulated in the RES group. Moreover, the expression pattern of these apoptotic proteins in the Com group closely resembled that observed in the RES group, but the effects were more pronounced (Figures 5D–H). These results demonstrate that RES induces substantial alterations in the expression of key regulatory proteins involved in apoptosis. Furthermore, while the combined treatment in the Com group exerts effects similar to those of RES alone, the magnitude of these effects is significantly enhanced. Collectively, these data support the conclusion that RES potentiates DOX-induced apoptosis in liver cancer cells. Changes in Bax/Bcl-2/caspase-3 signaling are correlative in the present study, and direct causal validation is beyond the scope of the current experimental design.

### 3.4 Protective effect of RES against DOX-induced myocardial injury

**FIGURE 6:**
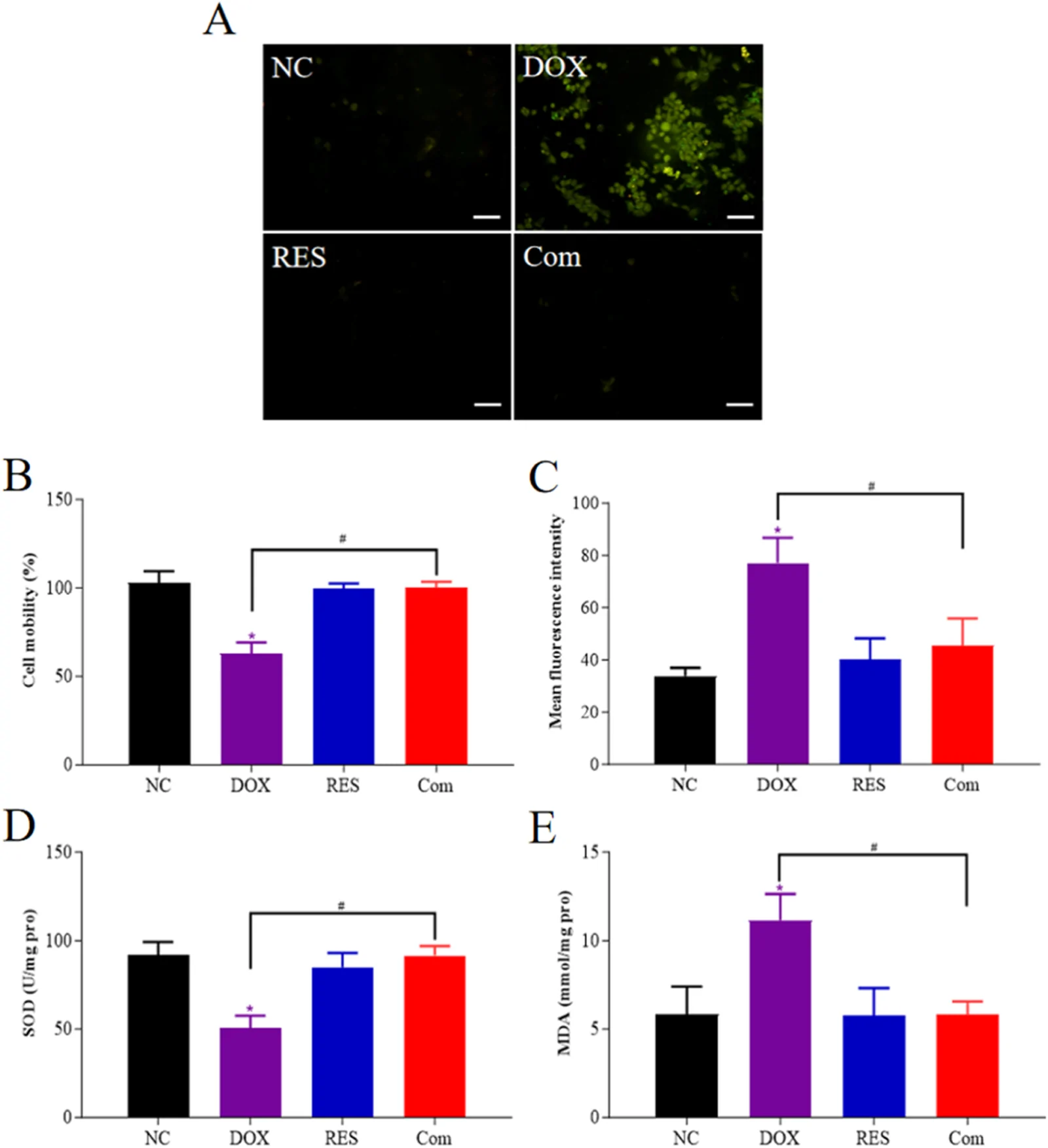
RES regulates DOX-induced oxidative stress injury in cardiomyocytes. **(A)** Representative ROS fluorescence staining images. **(B)** Cardiomyocyte viability after 24 h treatment. **(C)** Quantitative analysis of ROS fluorescence intensity. **(D,E)** Detection of SOD activity and MDA content. All assays were repeated three times biologically. Statistical analysis was performed via one-way ANOVA and Tukey´s multiple comparisons test. NC: Negative control; RES: Resveratrol; DOX: Doxorubicin; Com: RES + DOX. Scale bar: 50 μm *p < 0.05, **p < 0.01 vs. NC group; #p < 0.05, ##p < 0.01 vs. Com group.

In the myocardial cell damage model induced by DOX, exposure to DOX significantly impaired the viability of myocardial cells, resulting in a marked reduction in cell survival compared to the NC group. In contrast, pretreatment with RES effectively counteracted this detrimental effect, restoring cellular viability to a level close to the physiological baseline (Figure 6B). These findings suggest that RES exerts a direct antagonistic action against the cytotoxicity of DOX. Oxidative stress imbalance represents one of the central mechanisms underlying DOX-induced myocardial injury. Following DOX treatment, intracellular ROS accumulated rapidly, as evidenced by significantly elevated fluorescence signal intensity relative to the NC group (Figures 6A,C). Concurrently, the cellular antioxidant defense system was severely compromised-SOD activity declined sharply, while the levels of MDA, a terminal product of lipid peroxidation, increased dramatically. Together, these changes reflect a profound disruption of cellular redox homeostasis. RES intervention markedly reversed this pathological cascade: it not only suppressed the excessive production of ROS but also restored SOD activity to physiological levels and reduced MDA accumulation (Figures 6D,E). This comprehensive modulation of the oxidative damage-antioxidant defense-lipid peroxidation axis constitutes a key mechanism by which RES maintains redox equilibrium in myocardial cells and mitigates DOX-induced toxicity. The coordinated alterations observed at both phenotypic and molecular levels indicate that the protective effect of RES extends beyond a mere compensatory improvement in cell viability. Rather, by reestablishing oxidative stress balance, RES fundamentally alleviates DOX-induced oxidative damage in myocardial cells. These results provide mechanistic insights supporting the potential application of RES in the prevention and treatment of drug-induced cardiotoxicity.

### 3.5 Mechanism of myocardial cell apoptosis inhibition

**FIGURE 7:**
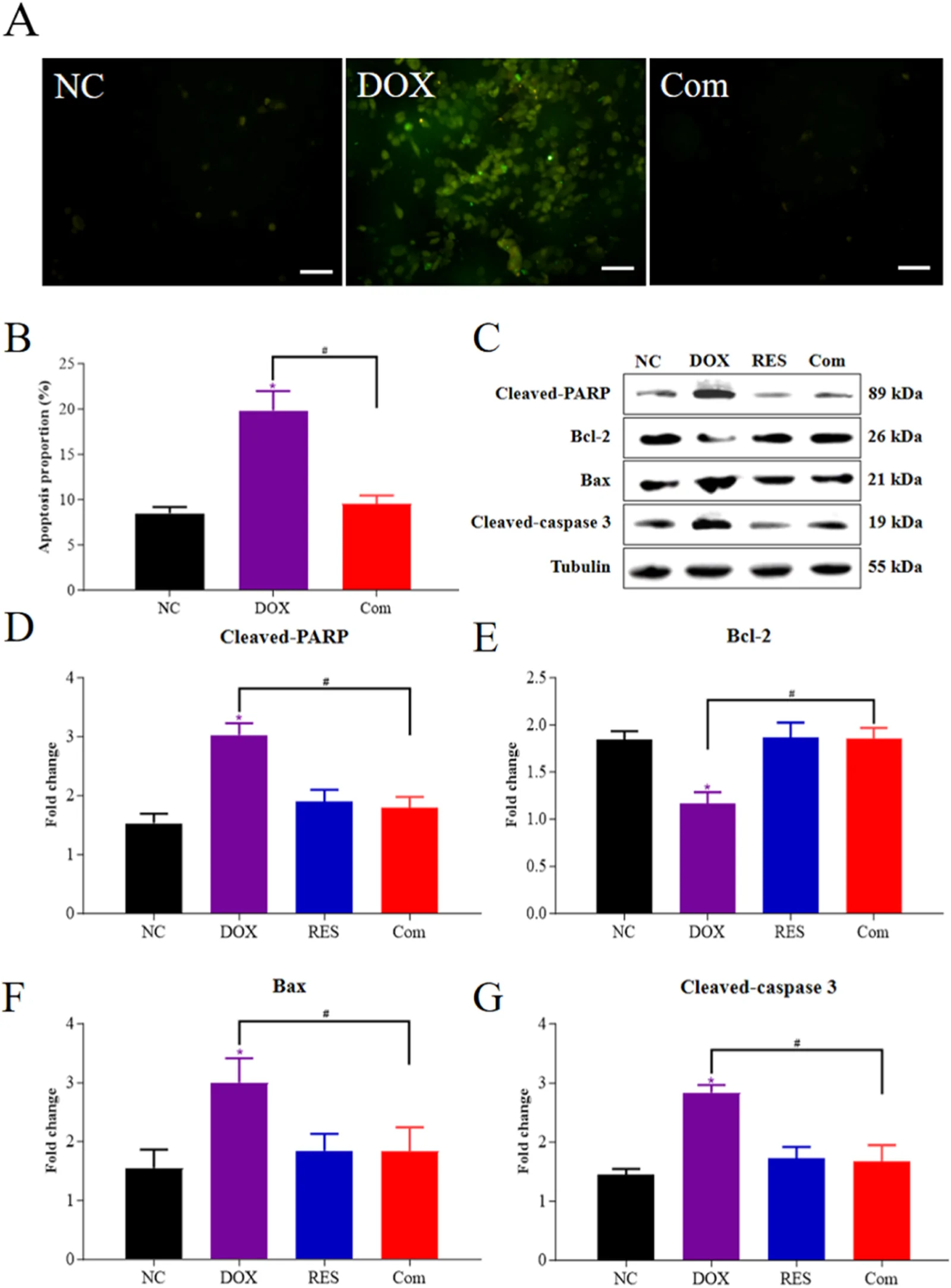
RES alleviates DOX-induced cardiomyocyte apoptosis. **(A)** TUNEL staining images of cardiomyocytes. **(B)** Statistical analysis of cardiomyocyte apoptotic rate. **(C–G)** Western blot detection and quantitative analysis of apoptosis-related proteins. All experiments were performed in three independent biological replicates. One-way ANOVA with Tukey´s *post hoc* test was used for statistical analysis. NC: Negative control; RES: Resveratrol; DOX: Doxorubicin; Com: RES + DOX. Scale bar: 50 μm *p < 0.05, **p < 0.01 vs. NC group; #p < 0.05, ##p < 0.01 vs. Com group.

In the DOX-induced model of myocardial cell apoptosis, TUNEL assay results demonstrated that apoptotic cardiomyocytes in the NC group exhibited almost no green fluorescence, indicating that the majority of cells remained viable and non-apoptotic. In contrast, following DOX exposure, a significant increase in green fluorescence signal was observed, suggesting that DOX potently activates the apoptotic program in cardiomyocytes. Notably, in the Com group, the green fluorescence signal was markedly reduced, the proportion of apoptotic cells decreased substantially, and the percentage of viable cells was restored to a level comparable to that of the NC group (Figures 7A,B). These findings provide direct evidence that RES exerts a potent inhibitory effect on DOX-induced myocardial cell apoptosis. Dysregulation in the expression of apoptotic regulatory molecules constitutes the core mechanism by which DOX promotes myocardial cell apoptosis.

Following DOX treatment, the expression of the anti-apoptotic protein Bcl-2 was significantly downregulated compared to the NC group, while the levels of the pro-apoptotic protein Bax were markedly upregulated. Furthermore, increased activation of key apoptotic executioner molecules-cleaved-caspase-3 and cleaved-PARP was detected. This collective shift establishes a pathological cascade characterized by impaired anti-apoptotic defense, enhanced pro-apoptotic signaling, and subsequent activation of the apoptotic execution pathway. RES intervention effectively restored this balance by normalizing the expression of the anti-apoptotic protein Bcl-2 to levels comparable to the NC group, while concomitantly suppressing Bax upregulation and significantly reducing the activation of cleaved-caspase three and cleaved-PARP (Figures 7C–G). This comprehensive modulation of the apoptosis regulatory proteins-apoptosis execution molecules axis represents a key mechanism through which RES inhibits DOX-induced cardiomyocyte apoptosis. The coordinated alterations observed from staining phenotypes to molecular expression profiles indicate that the protective effect of RES extends beyond a superficial improvement in cell survival. Rather, RES actively reshapes the expression balance of apoptosis-related molecules, thereby suppressing the activation of the apoptotic cascade from upstream initiators to downstream effectors. These findings provide dual experimental evidence at both phenotypic and molecular levels for the therapeutic potential of RES in mitigating DOX-induced cardiotoxicity.

### 3.6 Intracellular anti-tumor effect and myocardial protection

**FIGURE 8:**
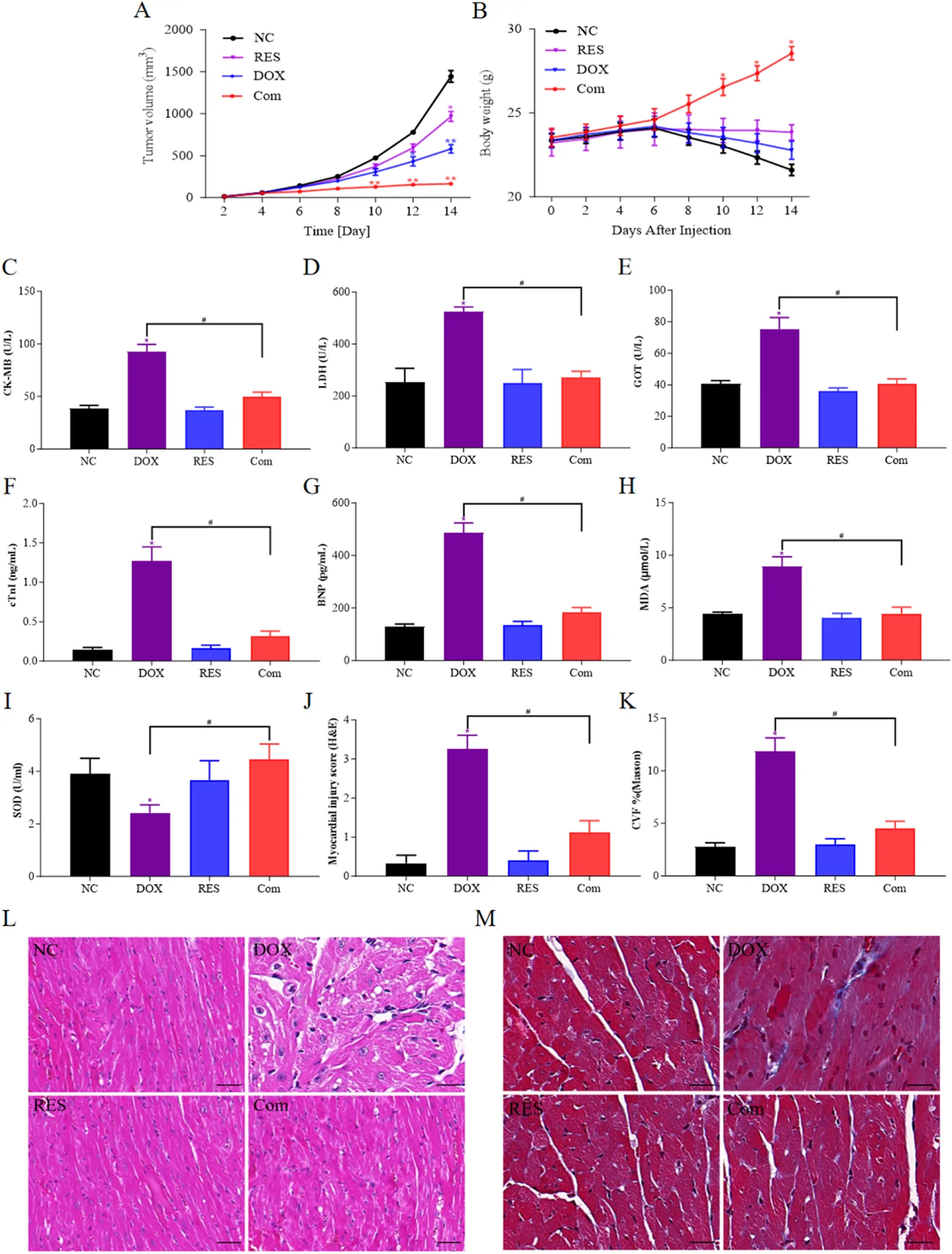
*In vivo* anti-tumor and myocardial protective effects of RES combined with DOX in H22 tumor-bearing mice. **(A)** Dynamic tumor volume curves. **(B)** Mouse body weight changes. **(C–G)** Serum CK-MB, LDH, GOT levels; MDA and SOD levels from myocardial tissue homogenates. **(H,I)** Serum cTnI and BNP levels. **(J,K)** Myocardial injury score and representative H&E staining images. **(L,M)** Myocardial CVF and representative Masson’s trichrome staining images. *In vivo* experiments included six mice per group (biological replicates). Statistical analysis was performed using one-way ANOVA followed by Tukey’s multiple comparisons test. NC: Negative control; RES: Resveratrol; DOX: Doxorubicin; Com: RES + DOX. Scale bar: 20 μm *p < 0.05, **p < 0.01 vs. NC group; #p < 0.05 vs. Com group.

Our *in vitro* cellular assays confirmed that combined RES and DOX treatment exerted prominent efficacy-enhancing combined inhibitory effects on the proliferation and metastasis of DOX-resistant HCC cells. To further verify the dual efficacy of tumor suppression and myocardial protection *in vivo*, a subcutaneous H22 hepatocellular carcinoma syngeneic mouse model was established. Tumor growth and mouse body weight were dynamically monitored throughout the 14-day treatment cycle. Meanwhile, multiple-dimensional indicators including systemic toxicity, serum clinical cardiac biomarkers, myocardial oxidative stress levels, and myocardial histopathological alterations were comprehensively detected to systematically evaluate the therapeutic advantages and biosafety of the combined regimen (Figure 8).

Consistent with the *in vitro* results, the *in vivo* tumor growth curve showed that DOX monotherapy significantly inhibited tumor proliferation, while RES monotherapy exhibited only mild and limited anti-tumor activity. The combination group achieved the strongest tumor suppressive effect, showing a significantly reduced tumor volume compared with the NC, RES, and DOX single-treatment groups. These results demonstrated that RES could effectively enhance the anti-tumor efficacy of DOX against resistant HCC, and importantly, RES co-administration did not antagonize the tumoricidal effect of DOX *in vivo* (Figure 8A). Body weight changes were recorded to evaluate systemic toxicity and treatment tolerance (Figure 8B). Mice in the NC group displayed continuous weight loss due to sustained tumor growth and nutrient consumption. The DOX monotherapy group showed significant body weight suppression starting from day 6, indicating severe systemic toxicity induced by DOX chemotherapy. In contrast, mice in the RES single group maintained stable body weight without obvious adverse reactions. Notably, the combination treatment effectively reversed DOX-induced weight loss, and mice in the Com group maintained steady body weight gain throughout the treatment period, suggesting that RES significantly improved the systemic safety and tolerability of DOX chemotherapy. To comprehensively validate the protective effect of RES against DOX-induced myocardial injury, we detected traditional myocardial injury enzymes (CK-MB, LDH, GOT), oxidative stress indicators (SOD, MDA), and supplemented clinical gold-standard cardiac biomarkers (cTnI, BNP), as well as myocardial histopathological staining. Biochemical detection results showed that DOX treatment significantly decreased myocardial SOD activity and increased MDA accumulation, resulting in severe oxidative stress imbalance. Meanwhile, DOX markedly upregulated the levels of CK-MB, LDH, and GOT, indicating obvious cardiomyocyte damage (Figures 8C–G). Importantly, RES intervention significantly reversed DOX-induced oxidative stress disorders, restored SOD antioxidant capacity, reduced MDA lipid peroxidation levels, and effectively decreased the release of myocardial injury enzymes. To further consolidate the evidence of myocardial protection, we supplemented the detection of serum cTnI and BNP, two core clinical biomarkers for evaluating myocardial injury and cardiac function. The results showed that DOX dramatically increased serum cTnI and BNP levels, strongly confirming severe cardiomyocyte necrosis and cardiac dysfunction caused by DOX. However, RES co-treatment significantly reduced the elevation of cTnI and BNP, effectively alleviating DOX-induced cardiac stress and myocardial damage (Figures 8H,I). H&E staining showed that the myocardium of NC and RES groups presented neatly arranged myocardial fibers, complete cell morphology, and no obvious inflammatory infiltration or tissue damage. In contrast, the DOX group exhibited typical pathological injuries, including disordered and broken myocardial fibers, cardiomyocyte vacuolar degeneration, nuclear pyknosis, and inflammatory cell infiltration, accompanied by a significantly increased myocardial injury score. After RES combined intervention, the myocardial tissue structure was significantly restored, the degree of cell edema and fiber disorder was greatly improved, and the myocardial injury score was significantly decreased (Figures 8J,K). Masson´s trichrome staining was further performed to evaluate myocardial interstitial fibrosis. The results revealed that NC and RES groups had minimal collagen deposition. DOX treatment induced extensive interstitial collagen hyperplasia and significantly elevated CVF, indicating obvious myocardial fibrosis. Notably, RES co-administration markedly reduced collagen deposition and decreased CVF values, effectively relieving DOX-induced myocardial fibrotic remodeling (Figures 8L,M).

Notably, RES monotherapy did not affect any oxidative stress indexes, myocardial injury biomarkers, or myocardial histological morphology, confirming that RES itself has no intrinsic cardiac toxicity. In summary, RES achieves dual regulatory effects *in vivo*: it enhances the anti-tumor efficacy of DOX on resistant HCC, and simultaneously ameliorates DOX-induced myocardial oxidative damage, structural pathological injury, and myocardial fibrosis via anti-oxidative and anti-fibrotic mechanisms. This dual-effect strategy significantly optimizes the therapeutic window and safety profile of DOX for drug-resistant hepatocellular carcinoma.

## 4 Discussion

This study addresses two critical clinical bottlenecks of DOX chemotherapy for liver cancer, namely, tumor drug resistance and dose-limiting cardiotoxicity. Via systematic *in vitro* and *in vivo* validation, we confirmed that RES could reverse DOX resistance in HCC cells to amplify the anti-tumor efficacy of DOX, and simultaneously alleviate DOX-triggered myocardial oxidative damage and apoptosis, achieving dual therapeutic effects of chemo-sensitization and cardiac protection. These findings indicate that RES achieves the triple therapeutic objectives of overcoming drug resistance, enhancing antitumor efficacy, and reducing cardiotoxicity. IC_50_ and RI detection verified the stable high DOX resistance of constructed cell lines, ensuring the reliability of subsequent mechanistic experiments.

The issue of drug resistance in liver cancer chemotherapy has emerged as the primary factor constraining patient survival benefits (Chan et al., 2024). Particularly among patients with advanced hepatocellular carcinoma, the high prevalence of DOX resistance poses a significant clinical challenge, leading to a progressive decline in the efficacy of conventional monotherapy (Berhane et al., 2022). These patients are confronted with the dual burdens of accelerated tumor progression and limited therapeutic options. The experimental findings of this study are highly consistent with clinically observed resistance patterns in the HepG2-R and SMMC-7721-R resistant cell lines: following 24 h and 48 h incubation with DOX alone, the inhibitory effect on the proliferation of resistant cells remains minimal, with cells maintaining robust adherence and exhibiting no significant differences compared to the normal control group. This strongly confirms that these resistant cells have developed complete tolerance to DOX, rendering monotherapy virtually ineffective in a clinical context. Monotherapy with RES exhibited mild antitumor activity. It slightly attenuated cell adhesion capacity and induced modest cellular damage. Mechanistically, RES moderately inhibited cell migration by downregulating the expression of mesenchymal markers (N-cadherin, vimentin) and core EMT transcription factors (snail, slug), while upregulating the epithelial marker E-cadherin.

These observations suggest that although the therapeutic potential of RES as a standalone treatment is limited, it possesses an intrinsic capacity to inhibit tumorigenic processes. Consistent with our results, previous studies have verified the chemo-sensitizing effect of RES on DOX-resistant multiple solid tumors. In DOX-resistant breast cancer, RES was proven to reverse chemotherapy resistance via inhibiting EMT and activating mitochondrial apoptotic pathways, significantly enhancing the cytotoxicity of DOX (Xu et al., 2017). Similarly, in drug-resistant osteosarcoma, RES combined with DOX could suppress tumor cell proliferation and metastasis, and block the PI3K/Akt drug-resistant signaling cascade (Chen et al., 2018). However, distinct from the single chemo-sensitizing effect reported in other DOX-resistant tumors, the present study further identifies the cell-specific differential regulatory characteristic of RES. Namely, RES sensitizes DOX-resistant HCC cells to chemotherapy while protecting cardiomyocytes from DOX-induced oxidative injury and apoptosis. This dual regulatory pattern has not been systematically elaborated in previous RES-related chemo-sensitization studies, which further highlights the unique superiority of RES in optimizing the safety and efficacy of DOX chemotherapy for drug-resistant liver cancer. Of note, although oxidative stress-related indices (ROS, SOD, MDA) were only measured in cardiomyocytes and myocardial tissues in our work, accumulating evidence indicates that mitochondrial remodeling and intracellular redox homeostasis also critically govern proliferation, migration, and drug-resistant phenotypes in hepatocellular carcinoma (Zhang et al., 2024). Mitochondrial network disturbance and redox imbalance are tightly intertwined with EMT reprogramming and apoptotic threshold shifts in HCC, implying that RES may also modulate tumor-intrinsic mitochondrial-redox signaling to contribute to its chemosensitizing effect, which warrants further experimental verification.

Notably, when RES is combined with DOX, a prominent combined efficacy-enhancing effect is observed-the inhibition rate of resistant cell proliferation is significantly enhanced, clearly demonstrating a favorable efficacy-enhancing interaction between RES and DOX. Morphological observations revealed that cells in the Com group exhibited markedly reduced adhesion capacity, a large number of suspended cells, a significant increase in damage-associated granular substances, and a sharp decline in cell density. At the molecular level, the Com group presented a robust combined regulatory pattern in the expression of EMT-related proteins: the expression level of the epithelial marker E-cadherin was significantly higher than that in the RES monotherapy group, while the expression of mesenchymal markers (N-cadherin, vimentin) and transcription factors (snail, slug) was further downregulated, with the most pronounced regulatory amplitude among all groups. Importantly, considering the significant cytotoxicity, proliferative suppression and cell detachment induced by the combined intervention concentration (40 μM RES +2 μM DOX) used in migration assays, the reduced wound healing rate and decreased transmembrane cell number in the combination group cannot be simply and solely attributed to specific EMT-mediated migration inhibition. Instead, the impaired migratory phenotype is a comprehensive result of non-specific cytotoxic cell loss, drug-induced proliferative arrest, and specific RES-mediated EMT pathway suppression. Despite the phenotypic confounding caused by drug toxicity, the consistent and robust modulation of EMT-related protein expression at the molecular level confirms that the combined treatment exerts definite specific regulatory effects on HCC EMT progression, rather than generating migratory inhibition merely through non-specific cell damage.

From the perspective of molecular mechanisms, RES can effectively reverse resistance to DOX and enhance its therapeutic efficacy, primarily by activating the apoptotic pathway and modulating the expression of proteins involved in metastasis. Apoptotic imbalance is widely recognized as one of the key contributors to the development of chemoresistance in tumor cells (Wu et al., 2017). In this study, DOX monotherapy showed no significant effect on the apoptotic pathways in HepG2-R and SMMC-7721-R cells. Specifically, there were no notable changes in the expression levels of the pro-apoptotic protein Bax, the apoptosis executioner molecules cleaved-caspase three and cleaved-PARP, or in the suppression of the anti-apoptotic protein Bcl-2, compared to the control group. In contrast, RES treatment alone only marginally upregulated the expression of Bax, cleaved-caspase 3, and cleaved-PARP, and exerted a limited inhibitory effect on Bcl-2. However, when DOX and RES were used in combination, a marked efficacy-enhancing effect on apoptotic regulation was observed, along with significantly enhanced suppression of Bcl-2. This pronounced bidirectional modulation of the pro-apoptotic and anti-apoptotic protein balance disrupted the apoptotic-resistant state of the resistant cells, thereby directly promoting their entry into programmed cell death.

Furthermore, tumor metastasis remains the primary contributor to the poor prognosis observed in patients with drug-resistant liver cancer (Huang et al., 2022). In this study, DOX monotherapy exhibited almost no inhibitory effect on the migration of resistant cells, while RES monotherapy only marginally reduced metastatic potential. Due to the inherent cytotoxic confounding factors of the therapeutic drug concentration, the anti-migratory phenotypic advantage of the combined treatment cannot be purely interpreted as specific migration suppression. Nevertheless, the significant remodeling of EMT-related proteins verified that RES can effectively strengthen the anti-metastatic molecular basis of DOX, thereby comprehensively curtailing the metastatic capacity of drug-resistant liver cancer cells through the superposition of cytotoxic clearance and targeted EMT regulation. This dual mechanism aligns well with the core therapeutic goals of suppressing primary tumor growth and preventing metastasis in the management of drug-resistant liver cancer cells.

In sharp contrast to the efficacy-enhancing effect observed in drug-resistant liver cancer cells, RES demonstrates a significant protective effect against DOX-induced cardiomyocyte injury, without compromising its antitumor efficacy. The dose-limiting cardiotoxicity of DOX remains a major challenge in its clinical application (van der Geest et al., 2025). In the H9c2 cardiomyocyte model, treatment with DOX alone was found to markedly reduce cell viability and induce severe oxidative stress imbalance, characterized by elevated levels of ROS, a significant decline in SOD activity, and a sharp increase in MDA content. This cascade establishes a vicious cycle of oxidative damage-impaired antioxidant defense-exacerbated lipid peroxidation. Furthermore, DOX potently triggers cardiomyocyte apoptosis, as evidenced by a pronounced increase in TUNEL-positive green fluorescence, with the proportion of apoptotic cells significantly higher than that in the NC group. At the molecular level, this is reflected in a pathological cascade involving a marked downregulation of the anti-apoptotic protein Bcl-2, upregulation of the pro-apoptotic protein Bax, and increased activation of cleaved-caspase-3 and cleaved-PARP.

Notably, RES intervention effectively reverses this cascade of damage: it restores cardiomyocyte viability to near physiological baseline levels, suppresses excessive ROS production, recovers SOD activity to normal physiological levels, and maintains MDA accumulation at a low threshold. Furthermore, RES restores the balance of apoptosis-related molecules by modulating their expression profiles. Specifically, it normalizes the expression level of Bcl-2 to that observed in the NC group, significantly suppresses the upregulation of Bax, and concurrently maintains low activation levels of cleaved-caspase three and cleaved-PARP. As a result, RES effectively blocks the apoptotic pathway from its upstream initiation to downstream execution. This protective mechanism-characterized by precise regulation of redox homeostasis and targeted inhibition of the apoptotic cascade-overcomes the limitations of conventional cardioprotective agents, which often compromise the anti-tumor efficacy of DOX. Importantly, when co-administered with DOX, RES achieves the dual therapeutic objectives of reversing drug resistance and enhancing anti-cancer efficacy while simultaneously reducing cardiotoxicity.

The mechanistic insights gained at the cellular and molecular levels were further corroborated by *in vivo* experiments using the H22 liver cancer syngeneic animal model, underscoring the translational potential of RES in clinical applications. The results from the *in vivo* studies demonstrated that, although DOX alone could significantly suppress tumor growth, its associated severe systemic toxicity and cardiomyopathy remain major concerns. Compared to the NC group, mice treated with DOX exhibited a significant reduction in body weight, decreased SOD activity in cardiac tissue, and elevated levels of myocardial injury biomarkers, including MDA, LDH, CK-MB, GOT, as well as clinical cardiac injury markers cTnI and BNP. Histologically, DOX caused prominent myocardial fiber disorganization, cellular degeneration and extensive interstitial collagen deposition. In contrast, the combination therapy of RES and DOX not only achieved significant tumor volume suppression but also improved systemic tolerability. Mice in the Com group maintained stable body weight gain, indicative of physiological homeostasis. Cardiac SOD activity was restored, and the levels of MDA, LDH, CK-MB, GOT, cTnI and BNP were markedly reduced, approaching near-physiological baseline levels-significantly lower than those in the DOX group. It should be emphasized that serum LDH, GOT and total CK-related enzymatic indicators are non-specific injury biomarkers and cannot independently serve as direct evidence for cardiac-specific damage. Therefore, we further adopted cardiac-specific biomarkers (cTnI, BNP), myocardial histopathological staining, together with oxidative-stress assessment to comprehensively evaluate myocardial protective outcomes, as recommended by recent pre-clinical cardiotoxicity assessment strategies (Yang et al., 2025). Direct assessment of mitochondrial homeostasis including mitochondrial membrane potential, respiratory metabolism, as well as cardiomyocyte-inflammatory cell crosstalk, together with emerging strategies such as engineered vesicle-based mitochondrial metabolic regulation, will be important directions for future cardioprotection studies (Christidi and Brunham, 2021). The present study focused on downstream redox imbalance and apoptotic phenotypes validated by ROS/SOD/MDA and apoptosis assays, and further dedicated mitochondrial functional tests will be performed in follow-up work. Consistently, H&E and Masson staining confirmed reduced myocardial injury scores and decreased collagen volume fraction in the Com group. Of note, RES monotherapy did not alter baseline levels of any cardiac oxidative or injury markers, nor did it induce pathological changes in myocardial tissue, indicating that RES itself exhibits no intrinsic cardiotoxicity and only exerts protective effects when co-administered with cardiotoxic DOX. These *in vivo* findings confirm that RES effectively preserves the anti-tumor activity of DOX while substantially mitigating its cardiotoxicity and systemic adverse effects. Notably, the H22 allograft mouse model adopted in this study was constructed with wild-type tumor cells without DOX-resistant phenotypic induction. Therefore, the *in vivo* experiments were only designed to verify the synergistic anti-tumor efficacy and cardiac protective safety of combined RES and DOX treatment, rather than to validate the *in vivo* drug resistance reversal mechanism. The core evidence for RES-mediated DOX resistance reversal is exclusively derived from *in vitro* resistant HCC cell models (HepG2-R, SMMC-7721-R). This outcome challenges the conventional notion that cardioprotection necessarily compromises anti-cancer efficacy, providing robust experimental evidence for achieving an optimal balance between therapeutic efficacy and safety in liver cancer chemotherapy.

The innovation of this study lies in its being the first to systematically elucidate the three-fold regulatory effects of RES on DOX-resistant liver cancer-namely, resistance reversal, efficacy enhancement, and myocardial protection based on the key experimental observations that DOX monotherapy is ineffective, RES monotherapy exhibits modest efficacy, and combination therapy demonstrates high effectiveness. This work systematically reveals the dual regulatory mechanism of RES on DOX-resistant liver cancer and DOX-induced myocardial injury at cellular and animal levels, providing preliminary experimental basis and theoretical reference for the development of RES-based adjuvant chemotherapy strategy for DOX-resistant liver cancer. The scientific value and clinical significance of this study are manifested in three primary aspects: First, it establishes the pivotal role of RES as a DOX resistance-reversal agent by reversing the resistant phenotype and thereby restoring the antitumor activity of DOX, offering a promising candidate for improving chemotherapy outcomes in patients with DOX-resistant liver cancer. Second, it uncovers the mechanism underlying RES-mediated antagonism of DOX-induced cardiotoxicity, primarily through modulation of oxidative stress and apoptotic pathways, thus providing a new protective strategy against the dose-limiting toxicities associated with anthracycline-based therapies. Third, through a comprehensive series of *in vitro* and *in vivo* experiments, the study confirms that RES achieves differential regulation-sensitizing tumor cells while simultaneously protecting myocardial cells-providing preliminary theoretical support for its subsequent preclinical transformation research. Furthermore, as a naturally occurring polyphenolic compound with abundant sources and a well-established extraction process, RES has been extensively studied and consistently shown to have low toxicity and minimal adverse effects. Consequently, the combination regimen of RES and DOX demonstrates favorable safety and feasibility, making it particularly suitable for patients with advanced DOX-resistant liver cancer or those requiring long-term, high-dose DOX treatment, and providing a preliminary experimental basis for subsequent preclinical research and regimen optimization.

This study has several limitations. First, the clinical translation of RES is limited by its inherent pharmacokinetic properties. As a natural polyphenol, RES exhibits poor oral bioavailability, rapid *in vivo* metabolic clearance, and low systemic bioavailability, which greatly hinders the maintenance of stable therapeutic concentrations in human circulation, thereby restricting its direct clinical application (Amri et al., 2012). Furthermore, the current study used intraperitoneal injection in mice, which differs from the oral or intravenous routes used clinically, leading to differences in *vivo* absorption, distribution, and effective exposure between animal models and human patients. In addition, this study lacks targeted exploration of the pharmacokinetic interaction between RES and DOX; whether RES regulates the tissue distribution, metabolism and excretion of DOX to exert dual efficacy remains unclear, which needs further verification. Second, although the drug-resistant cell lines and cardiomyocyte lines employed in the *in vitro* experiments can recapitulate key biological effects, they substantially differ from the complex tumor microenvironment and the *in vivo* metabolic conditions of the human body. Therefore, future studies should utilize patient-derived tumor organoids to further validate these findings. Third, the migration phenotypic experiments in this study have obvious confounding limitations. The drug concentration used for migration detection is the effective therapeutic concentration for reversing drug resistance and exerting synergistic anti-tumor effects, which inevitably induces cytotoxicity, cell proliferative inhibition and partial cell detachment in resistant HCC cells. These non-specific interfering factors cannot be completely distinguished from specific migration and EMT inhibition effects, leading to insufficient phenotypic specificity of *in vitro* migration results. Fourth, the optimal administration scheme of the combined regimen has not been systematically optimized, and the regulatory effect of RES on DOX pharmacokinetics needs in-depth investigation.

Future research may be directed along the following avenues: First, optimize the combination regimen of RES and DOX. Through comprehensive pharmacokinetic and pharmacodynamic studies, the optimal dosage, administration timing, and treatment cycle should be determined to maximize the therapeutic benefits of reversing drug resistance, enhancing efficacy, and reducing toxicity. Second, systematically investigate the shared and distinct molecular targets of RES involved in reversing drug resistance and providing myocardial protection. Multi-omics approaches-including transcriptomics, proteomics, and metabolomics-should be employed to construct differential regulatory networks in the context of drug-resistant hepatocellular carcinoma cells versus myocardial cells. Third, optimize the experimental conditions of cell migration assays, screen low-toxicity and non-lethal drug concentrations without obvious cell damage and detachment, and further verify the specific regulatory effect of RES on HCC migration and EMT progression under optimized experimental conditions to eliminate cytotoxic confounding factors and refine the anti-metastatic molecular mechanism. Fourth, conduct long-term follow-up in preclinical animal models to evaluate the effects of the combination therapy on liver cancer recurrence, metastasis, and long-term cardiac function. Finally, integrate clinical samples to validate the impact of RES on biomarkers of myocardial injury (e.g., troponin, brain natriuretic peptide) and indicators of tumor progression (e.g., tumor markers, imaging characteristics) in patients with DOX-resistant liver cancer undergoing chemotherapy, thereby facilitating the translation and clinical application of this therapeutic strategy from bench to bedside.

## 5 Conclusion

In summary, this study systematically investigated the triple regulatory mechanism of RES against DOX at cellular, molecular and animal levels. In DOX-resistant HepG2-R and SMMC-7721-R hepatocellular carcinoma cells, single DOX treatment showed negligible antitumor activity, while RES monotherapy only generated weak tumor suppression. The combined application of RES and DOX produced prominent efficacy-enhancing antitumor effects via activating apoptotic cascades and blocking EMT progression, which effectively reversed the chemoresistant phenotype. Notably, the suppressed migration phenotype of resistant cells induced by combined treatment is a comprehensive result of drug cytotoxicity, proliferative arrest and specific EMT inhibition, rather than pure specific migration suppression. In H9c2 cardiomyocytes, RES restored intracellular redox balance and inhibited apoptotic signaling to relieve DOX-triggered myocardial damage. The dual regulatory function of RES was fully verified in H22 syngeneic tumor-bearing mice: the combined therapy exerted an enhanced anti-tumor effect on the basis of retaining DOX´s antitumor activity while significantly alleviating systemic adverse reactions, serum myocardial injury, oxidative stress, myocardial structural lesions and interstitial fibrosis. This study provides reliable experimental basis for optimizing chemotherapy regimens for DOX-resistant hepatocellular carcinoma, and offers theoretical reference for applying natural polyphenols to overcome tumor chemoresistance, which provides a feasible experimental direction and theoretical reference for subsequent preclinical optimization of chemotherapy regimens for resistant liver cancer.

## Data Availability

The original contributions presented in the study are included in the article/supplementary material, further inquiries can be directed to the corresponding author.
